# Advent of robotic assistance in modern total knee arthroplasty (TKA): paradigm shift in orthopedic surgery

**DOI:** 10.1097/MS9.0000000000005455

**Published:** 2026-09-09

**Authors:** Iqra Akhtar, Ania Mushtaq

**Affiliations:** a Department of Medicine, Ayub Medical College, Abbottabad, Pakistan; b Department of Medicine, Mugda Medical College, University of Dhaka, Dhaka, Bangladesh

*Dear Editor,*

The advent of robotic assistance in total knee arthroplasty (TKA) heralds a paradigm shift in orthopedic surgery, offering unprecedented precision in implant positioning, superior radiographic outcomes, and enhanced long-term survivorship compared to conventional jig-based techniques^[^1^]^. With evolving modalities for rheumatoid arthritis care and growing global TKA volumes associated with an aging population, these systems address severe constraints in mechanical axis alignment and soft-tissue balancing, which are leading factors in premature failures, including instabilities and aseptic loosening, in up to 20 percent of instances^[^2^]^.

Robots such as MAKO (Stryker), ROSA (Zimmer Biomet), and OMNIBotics (Corin) have evolved into advanced systems that incorporate preoperative computed tomography (CT) or imageless registration, with haptic feedback, to provide real-time intraoperative guidance^[^1^]^. The wide-ranging review offered by Fan et al. explains that the systems are semi-autonomous and allow sub-millimeter accuracy in bone resection and component placement, minimizing mechanical axis outliers from 20% in manual TKA to 6% in robotic groups^[^3^]^. This precision promotes physiologic gap balancing and ligament tensioning, which form the basis of lasting kinematic restoration and can be assisted by AI-based models or augmented reality^[^4,5^]^.

Rigorous meta-analyses of randomized controlled trials (RCTs) substantiate these technical advantages with clinical correlates. Li et al. aggregated data from 20 RCTs encompassing over 2,000 patients, demonstrating robotic TKA’s superiority in postoperative coronal alignment outlier rate (5.4% vs. 23.6%; *P* < 0.001) and Knee Society Scores (mean difference: 4.2 points; 95% CI: 1.8–6.6, at 12 months)^[^6^]^.

Complementing this, Wang *et al*’s meta-analysis of 15 studies with an average of 10 years of follow-up found that robotic TKA has a higher survivorship rate than conventional techniques (96.2% vs. 92.8%, respectively), with a hazard ratio of 0.67 (*P* = 0.02), attributed to the maintenance of the joint lines and reduction of polyethylene wear^[^7^]^.

Adoption is also authenticated by patient-centered measures. The benefits of robotic TKA in the Oxford Knee Scores (standardized mean difference 0.31, *P* = 0.01) and a 15% absolute decrease in forgotten pain syndrome were confirmed by the evidence-based umbrella review of Garcia-Sanz *et al*, which synthesizes 12 previous meta-analyses and does not depend on the philosophy of alignment^[^3^]^. These are due to haptic constraints that prevent over-resection, as well as emergent artificial intelligence algorithms for predictive modeling of soft-tissues^[^8^]^.

Despite these strengths, there are barriers to implementation that should be examined. Capital investment is $1–2 million per unit, plus recurring disposables; however, health-economic models estimate that the cost is neutral, with decreased length of stay (0.5–1 fewer days) and revision rates^[^1,7^]^.

A steep learning curve (10–20 procedures) is a threat to residency training, which indicates the importance of simulation-based programs^[^8^]^. More importantly, level I evidence older than 10 years cannot be considered complete, and there is a risk that such evidence will be biased toward high-volume centers^[^3,6^]^.

Summarizing the main points, robotic TKA has evolved from an experimental adjunct to an indispensable precision modality that has already shown increases in alignment fidelity, functional recovery, and implant life.

The professional societies need to advocate for prospective registries, value-based payment models, and head-to-head comparisons, alongside newer, functionally aligned methods, to actualize their potential within the 2026 healthcare reforms. We invite readers of the journal to take part in this discussion, which will contribute to evidence-based innovation in arthroplasty.

This manuscript is compliant with the TITAN Guidelines and declares no use of AI^[^9^]^.

## Data Availability

Not applicable.
